# A malariometric survey of under-fives residing in indoor residual spraying-implementing and non-implementing communities of Lagos, Nigeria

**DOI:** 10.1186/s12936-016-1507-z

**Published:** 2016-09-07

**Authors:** Babatunde A. Odugbemi, Kikelomo O. Wright, Adebayo T. Onajole, Yetunde A. Kuyinu, Olayinka O. Goodman, Tinuola O. Odugbemi, Olumuyiwa O. Odusanya

**Affiliations:** 1 Department of Community Health & Primary Health Care, Lagos State University Teaching Hospital, 1-5 Oba Akinjobi Street, Ikeja, Lagos, Lagos State Nigeria; 2 Department of Community Health & Primary Health Care, Lagos State University College of Medicine, Lagos, Nigeria; 3 Department of Community Health and Primary Care, College of Medicine of the University of Lagos, Lagos, Nigeria

**Keywords:** Parasitaemia, Anaemia, Indoor residual spraying, Under-fives, Nigeria, Prevention, Morbidity

## Abstract

**Background:**

Indoor residual spraying (IRS) is used as part of the integrated vector management strategy for the control of malaria in Lagos, Nigeria. The purpose of this study was to compare the malariometric indices of children under 5 years old living in IRS-implementing and non-IRS-implementing communities of Lagos, Nigeria.

**Methods:**

The study was a community-based, comparative, cross-sectional study of 480 children under five recruited using a multi-stage sampling method. Data on each child were collected using a household questionnaire administered to the consenting care-giver of each selected child. Each child underwent a comprehensive physical examination. On-the-spot malaria rapid diagnostic testing and haemoglobin estimation to assess parasitaemia and anaemia, respectively, were also carried out. Risk factors for parasitaemia and anaemia were identified using multivariate logistic regression.

**Results:**

A total of 238 children were studied in the IRS-implementing group while 242 children were studied in the non-IRS-implementing group. The IRS -implementing community had a lower level of parasitaemia (1.3 %) compared to the non-IRS-implementing community (5.8 %) (*p* < 0.001). There was no significant difference in anaemia, spleen rate and fever in the IRS-implementing group (10.9, 9.7 and 5 %) and the non-IRS-implementing group (9.9, 8.8 and 8.7 %), respectively. Residing in an IRS-implementing community was associated with lower odds of parasitaemia (OR 0.17, *p* < 0.01). Sleeping under a bed net was the only factor associated with anaemia (*p* < 0.01).

**Conclusion:**

IRS has led to a reduction in the level of parasitaemia in the under-fives in the study areas.

## Background

Indoor residual spraying (IRS) is one of three main interventions recommended by the World Health Organization for the control of malaria. It is also one of the strategies recommended to achieve roll back malaria (RBM) updated targets. IRS is the application of long-lasting chemical insecticides on the walls and roofs of all houses and domestic animal shelters, in order to kill the adult mosquito vectors that come in contact with these surfaces. The intervention involves repeated spraying over a number of years [1, 2]. Nigeria has adopted it as part of its IVM strategy, however the use of ITNs is still the mainstay of Nigeria’s vector control strategy [1, 3].

In Nigeria, malaria constitutes a major public health problem as it has remained endemic despite control efforts by government and the civil society. Malaria is a preventable disease, affects all age groups, those particularly at risk are pregnant women and children, especially under-fives, and almost 110 million cases are reported every year. It is responsible for 60 % of outpatient visits to hospitals annually, 11 % of maternal deaths and 30 % of childhood deaths [4].

Varying results have been obtained from the different vector control methods used for the control of malaria in Nigeria [5]. Evidence from countries that have previously used IRS for malaria control suggest a rapid decline in malaria burden following introduction; this is shown in a review of the impact of IRS in six southern African countries [6]. Studies carried out in other locations, such as Malawi and Tanzania, also reported a lower prevalence of parasitaemia and anaemia in areas where IRS was carried out [7–9].

Lagos State, in addition to expanding coverage of ITNs, has embraced IRS as part of its IVM strategy in some local governments. Unlike ITNs that are individual-level interventions, IRS is a community-level intervention. There is limited information on the impact of IRS on malaria morbidity in vulnerable groups such as under-five children especially in high-burden countries such as Nigeria.

This study was a community-based, comparative cross-sectional survey which compared the malaria indices of under-fives in IRS-implementing and non-IRS-implementing communities in a district of Lagos, southwest Nigeria.

## Methods

### Study setting

Lagos State consists of 20 local government areas (LGAs), 16 of which are urban and four rural [10]. The LGAs are further divided into a varying number of wards. The study was carried out October 2014 in Iwerekun 1 and Orimedu 2 wards of Lagos. Both communities are located in Ibeju-Lekki LGA, which is a rural coastal district. The region is located within a tropical rainforest with two seasons, a rainy season (April–September) and a dry season (October–March). Transmission of malaria occurs throughout the year with peaks occurring in the rainy season.

Prior to this study, IRS with pyrethroid insecticides had been carried out in Iwerekun thrice over the previous 5 years with an estimated coverage of 80 %.

### Study population

The study population comprised children under the age of 5 years who had been resident in the community for a minimum of 6 months prior to the study and whose caregivers consented to participating in the study. Children who were acutely ill, determined based on information from care-giver or a medical report, were excluded.

### Sample size determination

A minimum sample size of 218 children in each community was calculated using the formula for the comparison of proportions for two independent groups [11] and based on a statistical power of 80 %, a level of significance of 5 % and ability to detect a 10 % difference in prevalence between IRS and non-IRS-implementing areas.

### Sampling technique

A multi-stage sampling technique was used for this study: in the first stage, one ward in which IRS was being implemented (Iwerekun 1) and another in which IRS was not being implemented (Orimedu 2) were selected by balloting; in the second stage, the houses in each of the selected wards were enumerated to determine the sampling frame for that ward and this was divided by the sample size to get a sample interval for each of the communities. Systematic random sampling was then used to select compounds consisting of one or more households. Finally, one household was selected in each compound by balloting. In each selected household, only one child under 5 years of age was selected. Where a household had more than one eligible child one was selected by balloting. If there was no eligible child in the selected household the next eligible house was selected.

### Study instrument and data collection methods

The instrument used was a pre-tested, structured, interviewer-administered household questionnaire, which was adapted from the Nigerian Malaria Indicator Cluster Survey [5].

### Interview

The questionnaires were administered to the mother of the child or the primary care-giver. One questionnaire was administered to one household in each compound. Care-givers were interviewed on demographic information, malaria preventive practices such as the use of ITNs, and history of febrile illness. The data were collected by trained interviewers and a laboratory technician.

### Physical examination

Each child had a physical examination, and weight and height measurements were taken. Axillary temperature was carried out using a self-calibrating digital thermometer to measure temperature closest to 0.1 °C. Any child found to be febrile was referred to the nearest health facility. Each child was also examined for splenomegaly by the researcher using Hackett’s method [12]. The child was made to lie flat on a hard surface and the abdomen examined for a palpable spleen. Where the spleen was palpable, it was measured using a tape measure. Finger-prick blood samples were taken from each child to assess parasitaemia and anaemia on the spot. Parasitaemia assessment was based on *Plasmodium falciparum* detection using malaria rapid diagnostic test (RDT) kits [First response malaria Ag*. P. falciparum* (HRP2) card test]. Haemoglobin concentration was assessed using a self-calibrating portable blood haemoglobin spectrophotometre (Hemocue Hb 301 haemoglobin system, Stockholm, Sweden). A new sterile lancet was used to make a single finger prick on the index finger of the non-dominant hand of each child after cleaning with alcohol swab.

### Outcome measures

*Parasitaemia* was defined as a positive RDT test result in the presence or absence of fever. *Anaemia* was defined as haemoglobin measurement of less than 11 g/dl [13]. Following examination, each child was classified as having a palpable enlarged spleen or not having a palpably enlarged spleen and the *spleen rate* for each community was calculated by dividing the number of children aged 24–59 months with enlarged spleens by the total number of children that were recruited into the study in the same age group. *Fever* was defined as an axillary temperature greater than 37.5 °C. The socio-economic status of each child was determined using a composite index based on the average score obtained on the occupation and highest educational attainment of both parents [14–16]. The social class ranged from 1 (highest) to 5 (lowest) [14]. The social classes were further grouped as upper class (social class 1 and 2), middle class (social class 3) and lower class (social classes 4 and 5). The higher occupational classes consisted of senior civil servants, medium to large-scale business owners and professionals while the lower occupational class comprised unemployed, petty traders and subsistence farmers. Parents’ educational achievements were ranked as high if they were graduates of tertiary institutions or had received professional training. Those with no formal education or educated to primary school level were ranked low. *Malnutrition* was defined as any child with weight for height (wasted), height for age (stunted) or weight for age (underweight) measurements <2 Z scores below the reference value [17].

### Data analysis

The data were analysed using SPSS version 20 software. Means and standard deviations were calculated for numerical variables and Student’s *t* test used to calculate the differences between means. Chi square tests were used to compare categorical variables as appropriate. Multiple logistic regression analysis was carried out to identify the factors associated with parasitaemia and anaemia. Level of significance was set at 5 % (*p* ≤ 0.05).

## Results

A total of 480 under-fives were recruited into this study, consisting of 238 and 242 in the IRS-implementing and non-IRS-implementing communities, respectively. The mean age of participants was 29.8 ± 15.9 and 26.8 ± 14.2 months in the IRS community and non-IRS-implementing community, respectively (Table 1). There was a higher proportion of males (56 %) in the IRS implementing community compared to the non-IRS-implementing community, which had a higher proportion of females (53 %). Mothers made up the majority of care-givers interviewed in both groups (95 vs. 95 %). The socio-economic status of the two groups was similar (*p* = 0.10) (Table 1).

**Table 1:** Sociodemographic characteristics of under-fives in both communities

Variable (child)	IRS (N = 238) Frequency (%)	Non-IRS (N = 242) Frequency (%)	χ2 *p* value
Age (months)			
Mean age ± SD	29.8 ± 15.9	26.8 ± 14.2	0.03*^#^
Gender			
Male	133 (55.9)	113 (46.7)	0.04*
Female	105 (44.1)	129 (53.3)	
Care-giver			
Father	6 (2.5)	6 (2.5)	0.95
Mother	226 (95.0)	231 (95.5)	
Other guardian	6 (2.5)	5 (2.0)	
Socio-economic status			
Upper class	96 (40.3)	75 (31.0)	0.10
Middle class	110 (46.2)	127 (52.5)	
Lower class	32 (13.4)	40 (16.5)	

^#^ *t* test *p* value

* p < 0.05

The prevalence of parasitaemia in the IRS-implementing community (1.3 %) was lower than in the non-implementing community (5.8 %) and this was statistically significant (OR 0.21, 95 % CI 0.06, 0.73) (Table 2). However, this was the only malariometric index that was significantly different between both groups. Both groups had a similar proportion with anaemia (IRS: 10.9 %; Non-IRS: 9.9 %), while the mean haemoglobin concentration of under-fives in the IRS-implementing community (16.3 ± 4.0 g/dl) was slightly higher than in the non-IRS implementing community (15.7 ± 3.6 g/dl) (*p* = 0.08). There was no statistical difference in the spleen rate among those aged 24–59 months in both groups (*p* = 0.91). At the time of the study 12 (5 %) under-fives were febrile in the IRS community while 21 (8.7 %) were febrile in the non-IRS community, but this difference was not statistically significant (*p* = 0.12). About a third of under-fives in both communities had slept under a bed net the previous night. Participants in both communities (IRS: 41.2 %; Non-IRS: 38 %) were reported to have had a fever in the 2 weeks prior to the study. These differences were not statistically significant (Table 2).

**Table 2:** Distribution of malariometric indices among under-fives in both communities

Variable	IRS (N = 238) Frequency (%)	Non-IRS (n = 242) Frequency (%)	χ2 *p* value	OR (95 % CI)
Parasitaemia	3 (1.3)	14 (5.8)	0.01**	0.21 (0.06, 0.73)
Anaemia	26 (10.9)	24 (9.9)	0.72	1.11 (0.62, 2.00)
Mean haemoglobin concentration (SD)	16.3 (4.0)	15.7 (3.6)	0.08^#^	0.35 (0.07, 1.29)
Splenomegaly (6–59 months)	23 (9.7)	21 (8.8)	0.71	1.13 (0.61, 2.09)
Spleen rate (24–59 months)	10 (7.0)	9 (6.7)	0.91	1.04 (0.41, 2.66)
Fever present	12 (5.0)	21 (8.7)	0.12	0.56 (0.27, 1.16)
Slept under net	90 (37.8)	79 (32.6)	0.24	1.25 (0.86, 1.82)
Fever in previous 2 weeks	98 (41.2)	92 (38.0)	0.48	1.14 (0.79, 1.65)

^#^Students *t* test p value

** *p* < 0.01

Table 3 shows the results of the logistic regression for parasitaemia and anaemia. Controlling for other variables, place of residence remained associated with parasitaemia (*p* = 0.01) with children residing in the IRS-implementing community having lower odds of parasitaemia (OR 0.17, 95 % CI 0.05, 0.62), while children who slept under a bed net the previous night had a lower odds of anaemia (OR 0.24, 95 % CI 0.12, 0.48). Living in an IRS-implementing area, recent history of fever, socioeconomic status and malnutrition were not associated with anaemia.

**Table 3:** Multiple logistic regression analysis of factors associated with parasitaemia and anaemia

Factor	Parasitaemia		Anaemia	
	*p* value	OR (95 % CI)	*p* value	OR (95 % CI)
Child age (months)				
6–12	0.16	0.19 (0.02, 1.87)	0.37	0.58 (0.18, 1.90)
13–24	0.29	0.46 (0.11, 1.94)	0.99	0.99 (0.33, 2.99)
25–36	0.37	0.53 (0.13, 2.11)	0.51	1.49 (0.46, 4.83)
37–48	0.13	0.25 (0.04, 1.49)	0.45	0.65 (0.21, 1.98)
49–59	Ref	1	Ref	1
Gender				
Male	0.56	1.36 (0.48, 3.86)	0.71	1.14 (0.58, 2.26)
Socio-economic status				
Lower	Ref	1	Ref	1
Middle	0.07	0.30 (0.08, 1.12)	0.65	1.23 (0.51, 2.94)
Upper	0.52	0.66 (0.19, 2.34)	0.16	2.08 (0.75, 5.77)
Child sleeping under bed net				
Yes	0.74	0.83 (0.27, 2.54)	0.00***	0.24 (0.12, 0.48)
History of fever				
Fever in previous 2 weeks	0.15	2.12 (0.75, 5.93)	0.21	0.65 (0.34, 1.27)
Malnutrition				
Yes	0.72	1.21 (0.42, 3.45)	0.46	1.31 (0.65, 2.62)
Place of residence				
IRS-implementing community	0.01**	0.17 (0.05, 0.62)	0.66	0.86 (0.44, 1.69)

** p < 0.01

*** *p* < 0.001

## Discussion

IRS was commenced in Lagos State in a bid for a more robust malaria control response. It is still a relatively new addition to the State’s vector control strategy and spraying has been carried out three times over the past 5 years. This study has shown that there was a lower risk of parasitaemia among children in areas where IRS was being carried out but the same did not apply to the other indices.

Although the study was carried out among apparently healthy children, an unexpected finding in was the relatively low prevalence of parasitaemia (Table 2). This was surprising due to the general classification of Nigeria as a high-transmission country and also the coastal geography of the study location, where vector breeding sites abound. This study was carried out in the month of October which corresponds with the start of the dry season. However, this is not likely to have affected the levels of parasitaemia as seasonality is not pronounced in the locality [18]. Other studies carried out in similar geographic and climatic settings have reported a higher prevalence, such as a malariometric study in Ikorodu area of Lagos and another in Oyo State in which 12.7 and 45.5 %, respectively, had parasitaemia [19, 20]. Another possible reason for the low prevalence observed in this study is the known over-diagnosis of malaria and the indiscriminate use of anti-malarial drugs in this environment [21, 22].

A consistent finding among studies carried out in other African countries comparing malariometric indices between areas of IRS-implementation and non-implementation is a significantly lower prevalence of parasitaemia, anaemia and splenomegaly in the IRS-implementing area compared to the non-IRS-implementing area [8, 9, 22]. In this study, there was a statistically significant difference in levels of parasitaemia alone. Although proportions of fever and anaemia were higher in the IRS-implementing area, the relationships were not statistically significant. The absence of any difference in fever between both groups is consistent with a malariometric study carried out to compare IRS and non-IRS-implementing communities in Malawi [8]. An explanation for the unexpected similar levels of anaemia, spleen rate and fever between the two groups could be that the use of IRS as a malaria control intervention is relatively new in the area. It is possible that with more frequent and sustained spraying a decline in the other malariometric indices would be observed.

Following multivariate analysis, this study showed that under-fives residing in IRS-implementing communities were less likely to have parasitaemia (*p* = 0.01) (Table 3). This suggests that IRS has been effective in reducing the transmission of malaria in the communities where it is being implemented and also supports findings from other studies in which there was a lower burden of malaria where IRS was carried out [8, 9]. An interesting finding was that sleeping under a bed net was independently protective of anaemia even when IRS was not (OR 0.24, 95 % CI 0.12–1.48). This could be because about one-third of under-fives in both IRS and non-IRS-implementing areas still slept under bed nets and bed nets have been used as a malaria preventive tool for a longer period than IRS. This finding suggests that bed nets should still be promoted even where IRS is being implemented.

The study limitation includes the cross-sectional study design which is not appropriate for making causal inferences due to the collection of exposure and outcome data at the same time. The causes of anaemia in this environment are multifactorial, however, this study did not investigate other factors that may affect anaemia, such as haemoglobinopathies, helminthiasis and HIV infection. The HRP-2 based RDTs used for the detection of parasitaemia in this study have a high sensitivity but are also reported to detect recently cleared infections in addition to current infections thus overestimating the prevalence of parasitaemia [23]. The performance of this type of RDT has however been validated in a similar setting [24].

Continuous efforts are needed to control malaria. As the prevalence of parasitaemia reduces, a robust surveillance system will be required to detect changes in transmission patterns. In addition, reducing transmission will also lead to reduced immunity, leaving the community more vulnerable in the event of sporadic outbreaks from imported cases.

Follow-up studies are required to confirm changes to malaria burden as malaria control efforts continue. Furthermore, longitudinal studies, which would allow calculation of malaria incidence and prove causal relationships, are required. Finally, entomological studies would add value to such comparisons. This will help in confirming the success of vector control programmes.

## Conclusion

This study shows parasitaemia was lower in the IRS-implementing community and this is likely to be due to the impact of IRS. Further studies are required to determine the long-term effect IRS on the burden of malaria in those areas.

## Abbreviations

IRS: indoor residual spraying
ITN: insecticide-treated bed net
IVM: integrated vector management
HRP2: histidine-rich protein 2
LGA: local government area
RDT: malaria rapid diagnostic test
RBM: roll back malaria
SES: socio-economic status
SR: spleen rate
WHO: World Health Organization
